# BARRIERS AND FACILITATORS TO DELIVERING PERSON-CENTERED NUTRITION FOR ASIAN AMERICANS IN ADULT DAY HEALTH SETTINGS

**DOI:** 10.1093/geroni/igz038.835

**Published:** 2019-11-08

**Authors:** Tina Sadarangani, Chau Trinh-Shevrin, Abraham Brody

**Affiliations:** 1 New York University Rory Meyers College of Nursing, New York, New York, United States; 2 New York university School of Medicine, New York, New York, United States

## Abstract

Malnutrition is a growing problem in community-based long-term care settings. Delivering person-centered nutrition is particularly important in congregate settings serving ethnically diverse older adults who have strong culturally-derived preferences around food. We conducted in-depth semi-structured multi-stakeholder interviews (N = 13) in an adult day health center (ADHC) serving Asian immigrants to explore the ADHC’s capacity to deliver person-centered nutrition interventions. Thematic analysis showed ADHCs successfully promoted social interaction at meal-time. However, participants had limited choice and restrictions on additives, like sodium, making it difficult to honor participants’ cultural preferences. Lack of flavor, limited choice, and rushed mealtimes, driven by center policies and procedures, disproportionately affected persons with dementia. Among those with dementia, clinicians disagreed whether nutrition should be used to manage chronic illness or whether a more palliative approach was warranted. One potential way to address this challenge would be to enable greater choice within a supportive ADHC mealtime environment.

